# Chromosomal instability and genomic alterations in cholangiocarcinoma from Northeastern Thailand

**DOI:** 10.1002/path.6464

**Published:** 2025-09-15

**Authors:** Raksawan Deenonpoe, Molly A Guscott, Sasithorn Watcharadetwittaya, Isabel L McNeill, Daniela Moralli, Nadeem Shaikh, Sarah E McClelland

**Affiliations:** ^1^ Barts Cancer Institute, Queen Mary University of London London UK; ^2^ Department of Pathology, Faculty of Medicine Khon Kaen University Khon Kaen Thailand; ^3^ Choligiocarcinoma Research Institute (CARI) Khon Kaen University Khon Kaen Thailand; ^4^ Nuffield Department of Medicine Oxford UK

**Keywords:** cholangiocarcinoma, liver fluke, chromosomal instability, heterogeneity, copy number alterations, single‐cell sequencing, Northern Thailand.

## Abstract

Cholangiocarcinoma (CCA) is a lethal cancer of the bile duct and is a major health concern in several parts of the world, including northeastern Thailand, where CCA incidence is the highest due to the endemic liver fluke *Opisthorchis viverrini*. Multiple studies have characterised genomic alterations in CCA tumours, and specific chromosomal alterations can predict prognosis. However, it is not known whether chromosomal instability (CIN), ongoing genomic alteration characteristic of most cancer types, is present in CCA tumours. In this study we leveraged a panel of cancer cell lines derived from fluke‐positive CCA patients, as well as a matched normal cholangiocyte line as a control, to characterise CIN in CCA. We observed elevated rates of chromosome segregation errors compared to normal cells, although overall CIN rates were lower than those for highly genomically unstable cancers, such as colorectal or ovarian cancer. Chromosome segregation errors in CCA cell lines were potentially driven by elevated DNA replication stress and centrosome duplication. Single‐cell genome sequencing and karyotyping of the cell lines showed extensive structural and numerical chromosomal aberrations, as well as copy number alterations that were heterogeneous between individual cells, supporting the presence of ongoing CIN in these cell line models. Low‐pass whole‐genome sequencing of 33 CCA tumour samples with matched normal tissue from northeastern Thailand, a liver fluke‐endemic region showed increased whole and subchromosomal level alterations, with a higher extent of genomic alterations in intrahepatic tumours compared to extrahepatic. Eight tumours carried focal amplifications and/or deletions involving known cancer genes, as well as potential chromosomal instability‐associated genes, including *CCNE1* amplifications and a rare amplification of *BRCA1*. This study provides increased understanding of the rate and potential mechanisms of CIN in CCA that may inform new therapeutic strategies that synergise with specific ongoing CIN mechanisms. © 2025 The Author(s). *The Journal of Pathology* published by John Wiley & Sons Ltd on behalf of The Pathological Society of Great Britain and Ireland.

## Introduction

Cholangiocarcinoma (CCA) is an aggressive heterogeneous epithelial cell malignancy that occurs along the biliary tree or within the hepatic parenchyma. CCA likely arises from epithelial cells lining the bile ducts. CCA may also develop from peribiliary glands and hepatocytes, depending on anatomical location and other factors such as duct type and MUC5AC levels [1, 2, 3]. The epidemiology of CCA varies globally, but is particularly prevalent in northeastern Thailand, which has the highest incidence of CCA in the world [4]. The incidence of CCA in the northern region of Thailand is 85 cases per 100,000 people, whilst in the southern region of Thailand there are 5.7 cases per 100,000 people [5]. Liver flukes, such as *Opisthorchis viverrini* and *Clonorchis sinensisare*, are a major risk factor for CCA in Southeast Asia [6], with several studies suggesting that liver flukes can exert carcinogenic effects though the induction of chronic inflammation in bile ducts during persistent infection [7, 8]. Incidence rates of CCA are 15 times higher in regions highly endemic for liver fluke infection than in areas where infection is rare [9]. For example, in Khon Kaen city in northeastern Thailand (an area endemic for liver fluke, with a population of 1.7 million), CCA is highly prevalent, with an age‐standardized incidence rate (ASR) of 58.8 and 23.6 per 100,000 males and females, respectively. However, Khon Kaen Cancer Registry data indicate declining CCA incidence between 2002 to 2013, and projections suggest stabilisation by 2025. Notably, this reduction in incidence mirrors the decreased prevalence of *O. viverrini* both locally and nationally, declining from over 60% in 1984 to less than 10% after 1997 [4]. Faecal analysis and the enzyme‐linked immunosorbent assay (ELISA) serve as diagnostic approaches for fluke infection. Nonetheless, it is worth noting that, based on serological testing, at least one‐fifth of CCA cases exhibit no prior chronic exposure to fluke infestation [10].

CCA is divided – based on anatomical position – into intrahepatic CCA (iCCA), perihilar CCA (pCCA), and distal CCA (dCCA). However, many databases categorise both pCCA and dCCA as extrahepatic CCA at the level of the cystic duct [11, 12, 13, 14]. CCA patients exhibit short survival times, even after receiving surgical treatment, since diagnosis often occurs when the disease has already reached an advanced stage, where the tumour has already progressed locally to involve adjacent vital structures [15].

Most solid tumours exhibit chromosomal instability (CIN), the ongoing acquisition of chromosomal alterations, leading to aneuploidy (gains or losses of chromosomes), and DNA copy number alterations (CNAs) [16, 17, 18]. CIN has been extensively associated with tumorigenesis, cancer progression, and may contribute to chemotherapy resistance [16, 18, 19]. CIN can originate from chromosomal segregation errors during mitosis, due to aberrant mitotic spindles, sister chromatid cohesion defects, or improper microtubule‐kinetochore attachments, among other causes [20, 21]. CIN may also be driven by DNA replication stress (stalled or collapsed replication forks) or defects in DNA repair. Demonstrating the presence of CIN as an ongoing phenomenon in patients is challenging, since the presence of chromosomal alterations in a tumour is indicative, but not conclusive, of CIN. Therefore, it is currently technically challenging to reliably distinguish between a short burst of instability and a prolonged period of ongoing CIN.

In previous studies of CCA tumours, genomic alterations detected using high‐resolution single‐nucleotide polymorphism (SNP) arrays revealed gains of chromosomes 1q, 8q, 17, and 20, and losses of chromosomes 1p, 3p, 6q, and 9 [22]. Additionally, patients with polysomy of chromosomes 7 and/or 17, as well as those with polysomy of chromosomes 3, 7, and 17 combined with 9p21 loss, had significantly shorter survival times compared to those without these chromosomal aberrations (median overall survival: 15.74 versus 37.57 months) [23, 24]. Additional studies have detected CNAs associated with CCA using whole‐genome sequencing [25, 26] (see Table 1 for a summary of all related studies). Whole‐genome and epigenetic profiling of liver fluke‐positive and liver fluke‐negative CCA patients identified four distinct clusters based on their genomic and epigenomic profiles, and indicated that fluke‐negative iCCA is associated with higher copy number alterations than fluke‐positive iCCA [25]. CCA has also been explored using single‐cell transcriptomics [33, 34]. One study also examined the CNAs inferred from single‐cell RNA sequencing in distal CCA and showed that tumours could be distinguished by 17p gains, or losses [33].

**Table 1:** Summary of previous studies identifying CNAs in CCA tumours, compiled from the following references: [25, 26, 27, 28, 29, 30, 31, 32]

Study	Lead author	Origin of samples	CCA subtype	Sample number	Fluke status incorporated	Chr aneuploidy gains; losses
Whole‐genome sequencing of 20 CCA cases reveals unique profiles in patients with cirrhosis and primary sclerosing cholangitis [28]	Holzapfel *et al* [28]	Canada	iCCA and eCCA (perihilar)	20	No	None; 3,4,6. (Authors also noted half samples are genome doubled)
Patterns of chromosomal copy‐number alterations in iCCA [29]	Dalmasso *et al* [29]	France	iCCA	53	No	1q, 7p, 7q,8q; 1p, 3p, 14q.
Whole‐genome and epigenomic landscapes of etiologically distinct subtypes of CCA [25]	Jusakul *et al* [25]	Thailand, Singapore, Korea, France, Romania, Brazil	iCCA and eCCA (perihilar)	71	Mixture of infected and non‐infected	Higher aneuploidy particularly of chr2 in fluke negative patients.
Integrative molecular analysis of iCCA reveals 2 classes that have different outcomes [30]	Sia *et al* [30]	Italy, Spain, USA	iCCA	153	No	1q,7p; 1p,3p,9p,9q,11p,13q,14q,1 7p,18q,21q
Development of a genomic predictive model for CCA using copy number alteration data [31]	Tavares *et al* [31]	Portugal	eCCA and iCCA	23	No	2q,6p,8p,17q; 4q,6p,Xp,Xq.
Whole exome sequencing of multi‐regions reveals tumour heterogeneity in tumour heterogeneity [32] *Opisthorchis viverrini*‐ associated cholangiocarcinoma	Sitthirak *et al* [32]	Thailand	unclear	13	Yes	5p,7p,8q,19q; 5q,9q,10p,13q,14q,17p,18 q,19p,21q,22q.
Common molecular subtypes among Asian hepatocellular carcinoma and CCA patients [26]	Chaisaing mingkol *et al* [26]	Thailand	iCCA	199	No	1p,1q, 2p,2q, 3p,3q,5p,6p,7p,7q,8p,8q,1 2p,13p,13q,14p,16p,16q, 17q, 19q, 20p,20q; 1p, 3p, 4p,4q,5q, 6q, 8p, 9p, 14q, 17p, 18p,18q, 19p, 21p,21q, 22q.
The genomic landscape of CCA reveals the disruption of post‐transcriptional modifiers [27]	Zhang *et al* [27]	China	iCCA	348	No	Focal amplifications at 7q31.2, 22q11.21; Focal deletions at 1p36.13, 2p24.1, 7q35, 12q24.33

Abbreviations: CCA, cholangiocarcinoma; iCCA, intrahepatic cholangiocarcinoma.

Increasing knowledge of targetable genetic lesions in CCA is informing new therapies [35]. However, to date, neither the definitive presence of CIN in CCA, nor the mechanisms that induce CIN have been determined in CCA. A previous study analysed gene expression data and predicted the role of defective mitotic spindle checkpoint activity in promoting high levels of aneuploidy in CCA [26]; however, functional studies have not yet been performed.

Temporal measurements (such as microscopy or tracking genomic alterations over time [36]) required to appropriately classify the presence of CIN are only possible to achieve using *in vitro* cultured cells. CIN can manifest as lagging chromatin, anaphase bridges, aneuploidy, and micronuclei formation, visible in dividing cells [18, 37, 38, 39]. In this study we focused on the functional and genomic characterisation of CIN phenotypes in CCA in order to improve our understanding of the molecular mechanisms that give rise to these complex chromosome aberrations in CCA cell lines and patient tumours. We focused on cell lines and tumour samples from fluke‐associated CCA and from fluke‐endemic regions of Northeastern Thailand, respectively, with a view to gaining a deeper understanding of the mechanisms of CIN arising in fluke‐associated CCA.

## Materials and methods

### Ethics approval

Ethics approval for the study was obtained from the Khon Kaen University Ethics Committee‐IRB for Human Research (No. HE651164).

### Cell lines

Three CCA cell lines, and one normal bile duct cell line, were maintained at 37 °C and 5% CO_2_. The cell line identities were confirmed by short tandem repeat profiling (ATCC/LGC Standards, Middlesex, UK). H‐69 is an SV40 large T‐antigen‐immortalised, morphologically normal biliary cell line obtained from the University of Edinburgh [40]. KKU‐M055 is a poorly differentiated tubular adenocarcinoma cell line derived from a male patient with hilar CCA. KKU‐100 is a poorly differentiated tubular adenocarcinoma, derived from a female patient with iCCA, while KKU‐213A was derived from a male patient with mixed papillary and nonpapillary adenocarcinoma. All patients were from *O. viverrini*‐endemic areas of Thailand. KKU‐100 and KKU‐213A cell lines were obtained from the Japanese Collection of Research Bioresources Cell Bank (JCRB, https://cellbank.nibn.go.jp/english), whilst H‐69 and KKU‐M055 were obtained from collaborators (see Acknowledgements).

KKU‐M055, KKU‐100, KKU‐213A were maintained in DMEM High Glucose (Merck, Hertfordshire, UK) with 10% FBS (Gibco ThermoFisher, Cheshire, UK) and 100 U penicillin/streptomycin (Gibco ThermoFisher). H‐69 was maintained in DMEM:F12 plus glutamax (Life Technologies, Cheshire, UK) with 5% FBS, 100 U penicillin/streptomycin, and essential supplements (1% MEM nonessential amino acids (Lonza, Little Chesterford, UK, BE13114E); 1% lipid mixture (Merck, L0288); 1% insulin‐transferrin selenium (Gibco, 51500–056); 0.01 g/ml Bovine Pituitary extract (Life Technologies, 13028‐014). Cells were routinely tested for Mycoplasma using MycoAlert PLUS Mycoplasma Detection Kit (LT07‐710, Lonza). Cells were passaged for a maximum of 12 weeks (12–14 passages).

### Immunofluorescence

Cells grown on coverslips were fixed with PTEMF solution (0.2% Triton X‐100, 0.02 mol/l PIPES [pH 6.8], 0.01 M EGTA, 1 mmol/l MgCl2, 4% formaldehyde). After blocking with 3% BSA, cells were incubated with the following primary antibodies, following the manufacturers' protocols: γH2AX (05‐636; Millipore/Merck); CREST (15‐234‐0001, Antibodies Incorporated, Davis, CA, USA); α‐tubulin (ab7291; Abcam, Cambridge, UK); Centrin‐3 (ab54531; Abcam), RPA (ab79398; Abcam). Secondary antibodies used were obtained from Invitrogen (Life Technologies, Cheshire, UK) and included: goat anti‐mouse AlexaFluor 488 (A11017), goat anti‐rabbit AF594 (A11012), and goat anti‐human AF647 (A21445). Coverslips were incubated with primary antibody for 1 h, followed by incubation with a secondary antibody for 30 min at room temperature. DNA was stained with DAPI (Roche, Indianapolis, IN, USA), and coverslips were mounted using Vectashield (Vector H‐1000, Vector Laboratories, Newark, NJ, USA).

### Microscopy

Analysis of chromosome segregation was performed in triplicate by microscopy, using at least 30 anaphase cells, 30 prometaphase cells, or 300 interphase cells, depending on the assay. Images were acquired using an Olympus DeltaVision RT microscope (Applied Precision, LLC, Seattle, WA, USA) equipped with a Coolsnap HQcamera (Applied Precision). Three‐dimensional image stacks were acquired in 0.2‐μm steps, using an Olympus 100× objective (Tokyo, Japan) for anaphase and prometaphase cells, or a 40× objective for micronuclei. Deconvolution of image stacks and quantitative measurements was performed with SoftWorx Explorer (Applied Precision).

### Metaphase spreads & M‐FISH

Cells at 80% confluence were incubated with 10 μg/ml colcemid (Gibco) for 2 h. Cells were then harvested, swelled with KCl (75 mm, 37 °C, 7 min) and fixed in 3:1 methanol: acetic acid. Metaphase spreads from each cell line were hybridised using the multiplex‐FISH (M‐FISH) probe kit 24XCyte (Zeiss MetaSystems, Milan, Italy), following the manufacturer's protocol. In brief, the slides were incubated at 70 °C in 2× saline‐sodium citrate (SSC) buffer (ThermoFisher) for 30 min, then allowed to cool at room temperature for 20 min. Following washing in 0.1× SSC, the cells were denatured in 0.07 mol/l NaOH for 1 min, then washed in 0.1× SSC and 2× SSC. The cells were dehydrated through an ethanol series, and air‐dried. The probe mix was denatured at 75 °C for 5 min and preannealed at 37 °C for 30 min. The total probe mix was applied to each slide, and a coverslip was placed on top. The slides were incubated at 37 °C for 3 days, then washed for 2 min in 0.4× SSC at 72 °C, followed by 30 s in 2× SSC with 0.05% Tween20 at room temperature, and finally mounted in DAPI/Vectashield (Vector Laboratories). Images were acquired on an Olympus BX‐51 microscope (Applied Precision) for epifluorescence equipped with a JAI CVM4þ progressive scanCCD camera (JAI) and analysed using the Leica Cytovision Genus (v. 7.1) software (Leica, Solihull, UK). Then 10–12 metaphases were karyotyped for each cell line.

### Single‐cell whole‐genome sequencing

Single cells were dispensed into 384‐well Lo‐Bind DNA plates (15911992; Eppendorf, Stevenage, UK) containing unique Nextera i5 and i7 primer pairs (Integrated DNA Technologies, London UK) using the CellenOne microfluidics platform (Scienion, Portsmouth, UK). Library preparation then followed the tagmentase based DLP+ method, as previously described [41]. For KKU‐M055, 384 single cells were seeded, and 284 single cells were successfully sequenced (50 were dropouts, so presumably no cell seeded, and 50 cells failed to generate enough coverage). Similarly, for KKU‐213A, 319/384 cells were successfully sequenced (with 55 dropouts and 10 cells giving poor coverage). Demultiplexing was performed using unique‐barcode identifiers with bcl2fastq (v. 1.8.4, Illumina [42]). Demultiplexed reads were trimmed using Trimmomatic [43], using Nextera adaptors and length quality parameters. Quality control was assessed using FastQC [44]. Trimmed reads were aligned to the GRCh38 human genomic reference using BWA‐MEM [45], and only reads over a map quality of 10 were used for downstream analysis. Copy number analysis was performed using AneuFinder (v. 1.26.0) [46] using 500 kb bins, GC‐content correction and blacklisting filtering. The eDivisive algorithm [46] was used to determine the most likely copy number states. Only cells with between 0.5–3 million reads/cell and verified even coverage across the genome were included, in order to ensure consistent CNA detection across all cells. CNAs below 0.8 Mb were excluded from further analysis. The fraction of genome altered (FGA) was calculated as the sum of the genomic segments that deviate from copy number 2, or deviate from mean ploidy, divided by the length of all segments, for each individual cell. Shannon entropy scores were calculated as described previously by Viznyuk [47].

Pseudobulks were generated by merging single‐cell BAM aligned files together for each tumour cell population, using SAMtools merge v. 1.19, [48]. Segmented copy number data from merged BAMs and bulk WGS BAMs were generated using QDNASeq [49], with bin sizes of 100 or 500 kb, followed by read counting, mappability correction, and GC content correction, as guanine and cytosine content can affect read alignment and distribution. Absolute copy number fitting was performed using ACE [50], using cellularity, ploidy, and error estimates from the *squaremodel* function.

### Copy‐number‐based phylogenetic clustering

Minimum consistent segmentation was performed on each single‐cell tumour population to normalise bin positions and to find the simplest segmentation (i.e. the fewest number of segments) that accurately represents the observed copy number data, which is required for MEDICC2 [51]. MEDICC2 was then applied using the total copy numbers function, while all other parameters were set to default.

### Formalin‐fixed, paraffin‐embedded (FFPE) tissue samples and cell lines extraction

A total of 33 FFPE CCA tissue samples were selected from the Pathology Service Unit, Srinagarindra Hospital, Khon Kaen University during the year 2020–2021. Each FFPE tissue block was sectioned to obtain four 10‐μm slide‐mounted tissue sections. A board‐certified pathologist identified and demarcated cancerous and noncancerous areas on annotated slides, ensuring a minimum tumour content of 60% of the whole tissue section for reliable genomic analysis. Tumour and adjacent normal sections were excised from the marked areas using a manual macrodissection technique, with a new scalpel used between each sample to prevent cross‐contamination, and placed into microtubes. DNA extraction was performed using the QIAamp DNA FFPE Advanced UNG Kits with Buffer LF (QIAGEN, Manchester, UK). DNA was extracted from CCA and normal bile duct cell lines using the Puregene® cell kit (QIAGEN, Germantown, MD, USA) for high molecular weight gDNA extraction. DNA yield averaged 50–400 ng/μl, and 260/280 ratios ranging from 1.8 to 2.0, as measured using a Qubit instrument (Qubit4 Q33226, Invitrogen) and a Nanodrop instrument (Nanodrop 2000, ThermoFisher) for sample quality. The size and DNA integrity number were evaluated using genomic DNA ScreenTape® on the TapeStation platform (Tapestation 4200, Agilent Technologies, Didcot, UK) with TapeStation Analysis Software (v. 4.1.1; Agilent Technologies, 2021).

### Ultra‐low pass whole genome sequencing

Sample processing and whole‐genome sequencing (WGS) were carried out by The Institute of Cancer Research (ICR), Sutton, UK. Samples were pooled in equimolar ratios and sequenced to a depth of 1–2 million reads per sample using the Illumina® NovaSeq S1 PE100bp platform (Illumina, Cambridge, UK). Bioinformatics analysis was performed at the Queen Mary University of London (QMUL) High Performance Computing Cluster. Demultiplexed reads were trimmed using Trimmomatic [43] using TruSeq adaptors (Illumina) and length quality parameters and quality control was performed using FastQC [44]. Trimmed reads were aligned to the GRCh38 genomic reference using BWA‐MEM, with only reads over a map quality of 10 used for downstream analysis. The QDNAseq R package [49] was used to bin aligned reads using 500 kb bins, followed by GC correction and blacklisting. To call copy numbers, the R package ACE was used [50]. A cellularity of 0.3 was selected based on ACE predicted purity estimations, which were centred around 0.3 when assuming a diploid ploidy of *n* = 2 (supplementary material, Figure S1A). To verify that this choice of cellularity was not generating artefacts, DNA copy number alterations were mapped using a range of cellularity values between 0.1 and 1, confirming that profiles were qualitatively unchanged, irrespective of the cellularity used (supplementary material, Figure S1B). For adjacent normal samples, a threshold of 0.3 was selected to detect potential subclonal CNAs, and to match the tumour threshold.

### Statistical analyses

Student's *t*‐tests were used to identify significant differences between H‐69 and individual CCA cell lines, with standard nomenclature used, as noted in the figure legends. These were calculated using Prism software (v. 10.3.0, Graphpad, Boston, MA, USA). Independent Student's *t*‐tests were calculated to identify significant differences between KKU‐213A and KKU‐M055, and between iCCA and distal CCA FFPE tumours samples (extrahepatic and perihilar), as noted in the figure legends using the SciPy [52] statistical functions.

## Results

### Ongoing chromosomal instability (CIN) in CCA cell lines is likely driven by premitotic defects and supernumerary centrosomes

**Figure 1:**
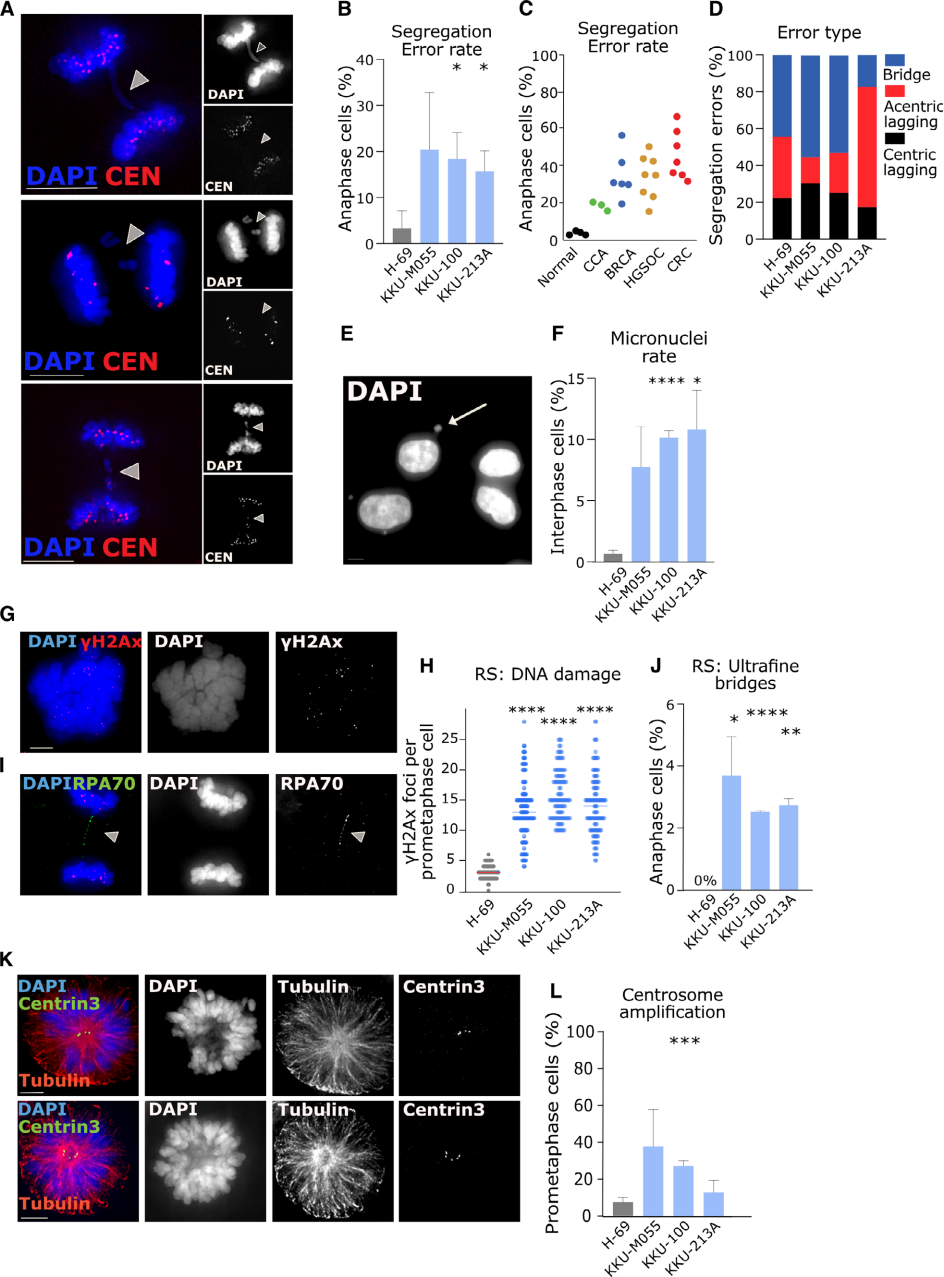
Evidence of chromosomal instability in fluke‐associated CCA cell lines. (A) Representative immunofluorescence (IF) images of CCA cells undergoing anaphase, with examples of KKU‐100 cells exhibiting a chromatin bridge, lagging acentric fragment or lagging centric chromosome. Scale bar, 5 μm in this and all other images. (B) Segregation error rates in cell lines as indicated, mean ± SD of three experiments (*n* = 120, 83, 113, and 115 cells scored in total for H‐69, KKU‐M055, KKU‐100, KKU‐213A cell lines, respectively). Student's *t*‐tests were used to test significance between normal H‐69 and each CCA cell line (*p < 0.05; **p < 0.005; ****p < 0.00001; no asterisk indicates *p* > 0.05). (C) Summary of segregation error rates of cell lines from different cancer types and normal cell lines (taken from [18, 39, 53]. Each dot represents error rates derived from one cell line of that type. The ‘normal’ cell line data are derived from H‐69 (this study), hTERT‐RPE1 and FNE1 epithelial cell lines or BJ‐TERT fibroblasts. (D) Segregation errors from (B) classified into error type (*n* = 4, 20, 19, and 19 errors scored in total for H‐69, KKU‐M055, KKU‐100, KKU‐213A cell lines, respectively). (E) Example image of KKU‐100 interphase cells, with arrow indicating the presence of a micronucleus. (F) Micronuclei rates in cell lines as indicated, mean ± SD of three experiments (*n* = 921, 786, 766, and 1,007 interphase cells scored in total for H‐69, KKU‐M055, KKU‐100, KKU‐213A cell lines, respectively. (G) Example immunofluorescence image of KKU‐213A prometaphase cell stained using antibodies for γH2Ax. (H) Quantification of DNA damage foci in prometaphase cells as a proxy measurement of replication stress (RS). Summary of three experiments (*n* = 121, 120, 107, and 119 cells scored in total). (I) Representative immunofluorescence image of KKU‐100 anaphase cell with ultrafine bridge stained using antibodies against RPA70. (J) Quantification of ultrafine bridges; mean ± SD of three experiments (*n* = 123, 69, 112, and 108 anaphases). (K) Panel of immunofluorescence images of KKU‐100 prometaphase cells arrested for 2 h with STLC (10 μm) and stained for microtubules and centriole pairs to demonstrate cells with normal (top) or amplified (bottom) centrosomes. (L) Quantification of prometaphase cells with more than four centrioles; mean ± SD of three experiments (*n* = 120, 111, 116, and 104 cells).

To determine whether CIN is present in CCA, we analysed three established CCA cell lines – KKU‐M055, KKU‐100, and KKU‐213A – all originally derived from Thai patients from fluke‐endemic regions (see Materials and methods). These were studied alongside a normal bile duct cell line, H‐69, using a panel of cell biological assays that we have previously used to assess CIN rates and mechanisms in other cancer types [18, 39, 53]. First, we analysed the rate of chromosome segregation errors in anaphase cells (Figure 1A,B). Segregation error rates in normal H‐69 cells were low (between 0% and 5% anaphase cells), close to what has been observed in other nontransformed, immortalized cell lines such as hTERT‐RPE1, BJ‐TERT, or FNE1 [18, 54], whereas in the CCA cell lines, the error rate was close to 20% of cell divisions (Figure 1B). This suggests that CCA does exhibit CIN, although the segregation error rates are relatively low compared to the rates previously observed in cancer cell lines from other tissue types such as high‐grade serous ovarian, colorectal, or breast cancers [18, 36, 39, 53] (summarised in Figure 1C). Next, we classified the segregation error subtypes, since these can be suggestive of the mechanism driving instability [18, 39]. Using CREST antibodies to mark the centromere, we classified segregation errors as either lagging chromosomes (centromere positive or negative) or chromatin bridges (Figure 1D). Centromere‐positive lagging chromosomes comprised a minority of errors across all cell lines (~20%–30%), whereas acentric fragments or chromatin bridges were more common (~70%–80%), suggesting that defects in DNA replication or repair were more prevalent than mitotic defects (such as cohesion fatigue, merotelic attachments, or spindle assembly checkpoint issues), consistent with observations in other cancer types we have analysed [18, 39, 53]. As mitosis completes, lagging chromosomes are often encapsulated into discrete structures separate from the main nucleus, called micronuclei [55]. Micronuclei rates were therefore scored as an additional indicator of CIN, and these were again higher in the CCA cell lines than H‐69 (around 5%–15% in cancer cells compared to ~1% in H‐69) (Figure 1E,F).

Given the prevalence of different classes of segregation errors, we looked for further evidence of mechanisms driving those errors. Previous work has suggested that when DNA replication is perturbed and cells suffer from DNA replication stress, then there is a greater incidence of acentric segregation errors during mitosis [39]. One proxy marker for replication stress is the presence of double‐strand breaks in fragile, underreplicated DNA (identifiable by the histone marker γH2Ax) in prometaphase cells, as DNA condenses in preparation for cell division [39, 56]. We quantified γH2Ax foci and observed significantly higher levels in all three CCA cell lines compared to H‐69 (Figure 1G,H). In parallel, we investigated the presence of ultrafine DNA bridges (UFBs) in anaphase cells, which can form when underreplicated DNA becomes entangled and remains unresolved as cells progress into anaphase [57]. UFBs can be detected by immunostaining for proteins that bind to single‐stranded DNA regions, using antibodies against BLM or RPA70 proteins [57]. Again, the presence of these structures was significantly higher in all three CCA cell lines compared to H‐69 (Figure 1I,J), although notably lower than levels previously observed in other cancer types [18, 39, 53]. Together, this evidence suggests that a moderate level of DNA replication stress is present in CCA cell lines and is therefore a potential driver of CIN in this disease.

Centrosomes are the main microtubule nucleating centres of mammalian cells, and their number is tightly regulated in normal cells to four centrioles (two per centrosome). Amplification of centrosomes is common in many cancers and can be related to chromosomal instability by increasing merotelic attachments *via* multipolar spindles [58, 59]. We scored for the presence of more than four centrioles in prometaphase cells and found that the proportion of cells with over four centrioles was higher in all three CCA cell lines, although only reaching significance in KKU‐100 compared to H‐69 (Figure 1K,L).

### Highly rearranged and heterogeneous genomes indicate ongoing chromosomal instability in CCA cell lines

**Figure 2:**
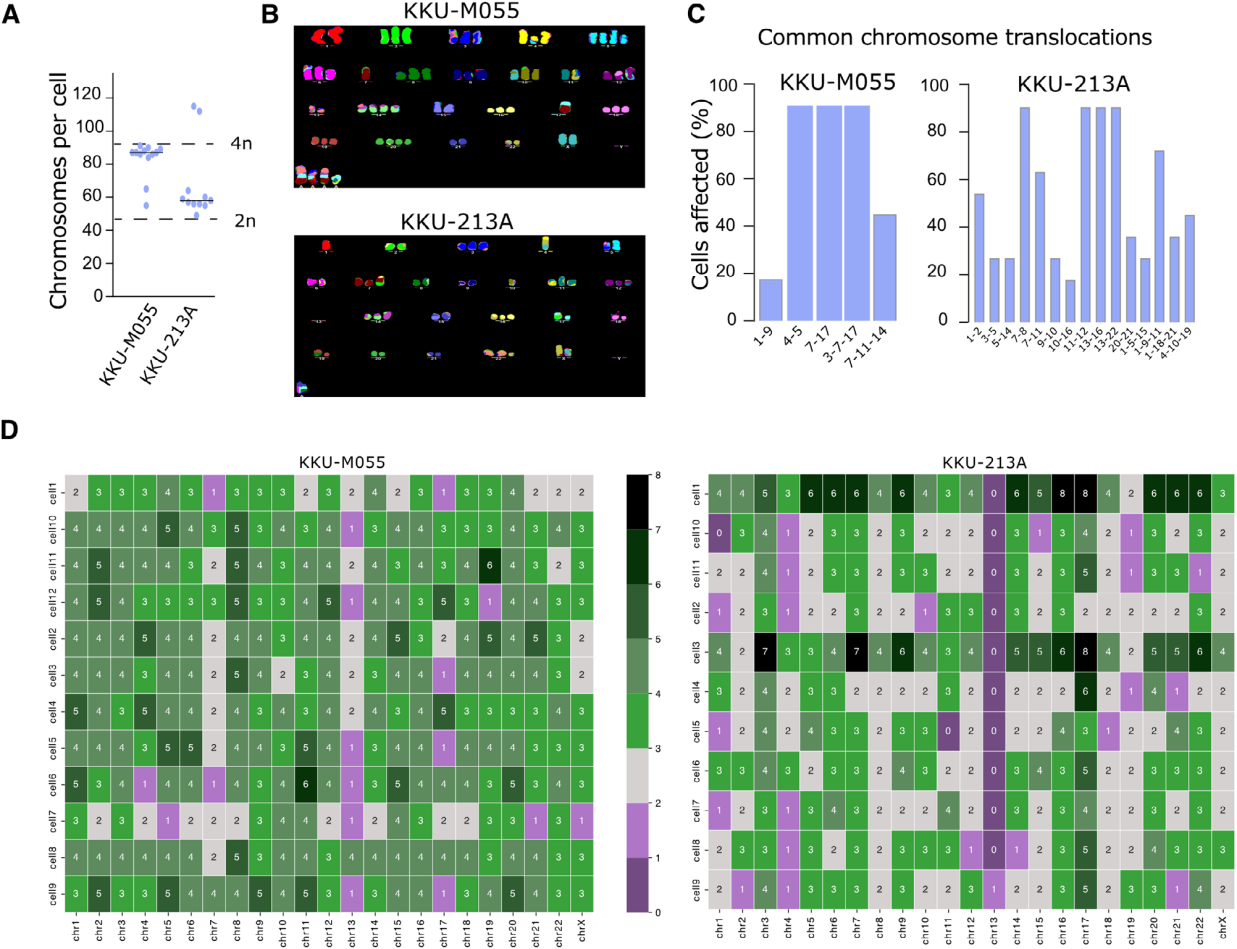
Chromosomal instability (CIN) promotes aneuploidy and heterogeneity in cholangiocarcinoma (CCA) as evidenced by metaphase spread analysis. (A) Quantification of chromosomes per cell from 11 to 12 metaphase spreads taken from two CCA cell lines. (B) Example images of mFISH metaphase spreads from KKU‐M055 and KKU‐213A. (C) Analysis of mFISH data showing frequency of the most common translocations in two cell lines analysed. (D) Heatmap indicating chromosome copy number changes quantified from KKU‐M055 and KKU‐213A mFISH images.

Ongoing chromosome mis‐segregation leads to increased numerical and structural defects at the chromosome level. We therefore investigated the consequences of CIN on the genome using multiplex FISH (mFISH) to estimate the ploidy of each cell line, by counting chromosome numbers from each cell. While KKU‐M055 ploidy was close to tetraploid, KKU‐213A was closer to hyperdiploid (Figure 2A). mFISH also revealed high rates of structural rearrangements, and widespread cell‐to‐cell heterogeneity in both cell lines tested (Figure 2B–D; supplementary material, Figure S2). A small proportion of higher ploidy cells was also noted in the KKU‐213A cell line (Figure 2A,D).

**Figure 3:**
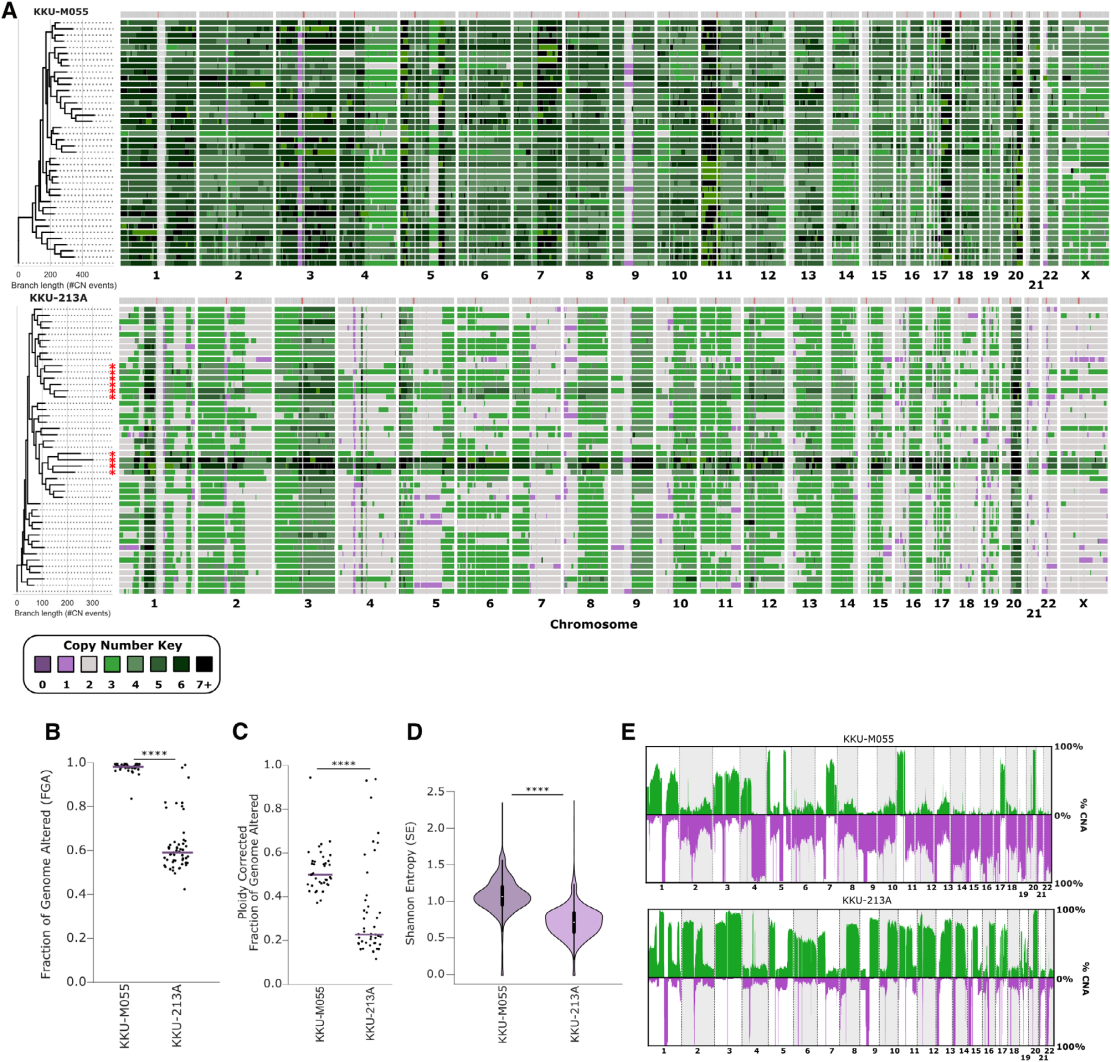
Chromosomal instability (CIN) promotes copy number alterations in cholangiocarcinoma (CCA) cell lines. (A) Low‐pass whole‐genome sequencing copy number heatmaps for KKU‐M055 and KKU‐231A, as indicated. Each row represents a single cell, each column represents a chromosome, and the colour (indicated by key) represents different copy numbers. Red asterisks indicate cells with a higher defined baseline ploidy, which were used to create tetraploid pseudobulk profiles in supplementary material, Figure 3C. (B–D) From the single‐cell sequencing data, we calculated the fraction of genome altered (B), fraction of genome altered corrected for mean ploidy of the population (C), and Shannon Entropy, a diversity metric (see Materials and methods) (D). (E) CNA frequency plots for the cell lines calculated from pseudobulks of the single‐cell data. Independent Student's *t*‐test used to test significance between KKU055 and KKU213 (*p < 0.05; **p < 0.005; ****p < 0.00001; no asterisk indicates *p* > 0.05).

Given the level of heterogeneity evident from the mFISH analysis, we proceeded to use single‐cell whole‐genome sequencing, adapting the DLP‐plus protocol [41] (see Materials and methods) to visualise cell–cell heterogeneity. Additionally, to our knowledge, single‐cell whole‐genome sequencing has not been previously performed in CCA. We sequenced both KKU‐M055 and KKU‐213A cell lines (Figure 3A, supplementary material, Figure S3). As with our mFISH data (Figure 2A), KKU‐M055 showed evidence of whole‐genome doubling. We also found evidence of widespread cell–cell heterogeneity, with KKU‐213A showing possible signs of at least three distinct subclones. To formally define subclones we used MEDICC2 [51] to perform CNA‐based phylogenetic clustering. Both cell line populations clustered into three or four major clades (Figure 3A). In KKU‐213A, two smaller clusters appeared to be distinguished on ploidy differences, consistent with the mFISH data (Figure 2A). To further investigate this finding, we produced pseudobulk CNA profiles of these subclones (see Materials and methods, and supplementary material, Figure 3C), which demonstrated very similar CNA profiles between the higher, and lower ploidy subclones, apart from a small region on chromosome 5q. We also noted that the Fraction of Genome altered (FGA) and overall heterogeneity (measured by Shannon Entropy, see Materials and methods section) were both significantly higher in KKU‐M055 compared to KKU‐213A cells (Figure 3B–D), although both cell lines exhibited highly chaotic genomes when the genome‐wide CNA frequency across all cells was analysed (Figure 3E).

### A Northeastern Thailand‐specific CCA cohort exhibits recurrent copy number alterations, including focal amplifications

**Figure 4:**
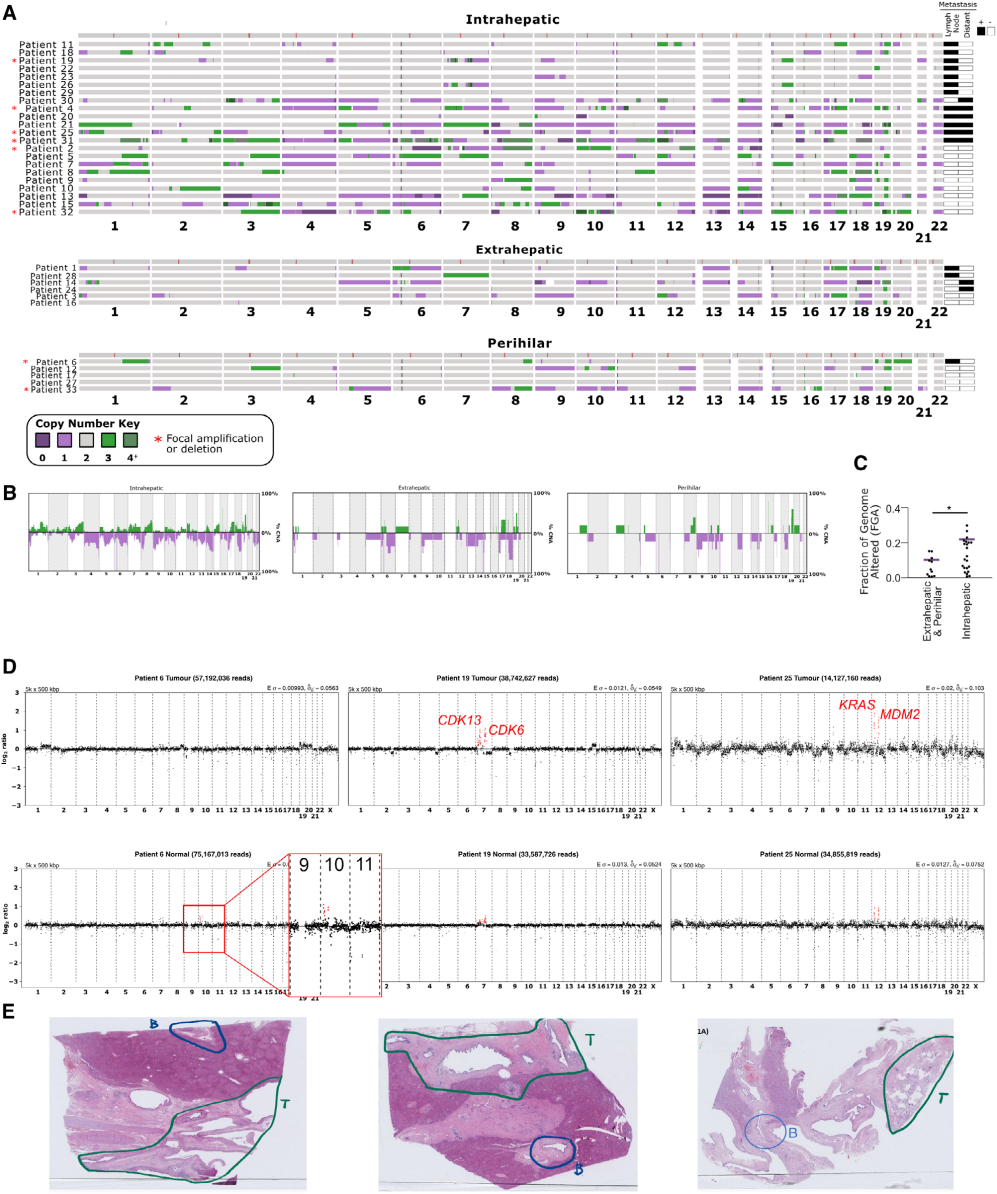
Genomic alterations detected from cholangiocarcinoma (CCA) tumours. (A) Copy number alteration heatmaps of FFPE tumour samples, grouped by CCA subtype. Each column represents a chromosome, and the colour (indicated by key) represents a different copy number. Tumours that exhibit focal amplifications are indicated with red asterisks (Figure 5; supplementary material, Table S3). Metastasis status of each patient indicated to the right of the heatmap. (B) Copy number frequency plots of each CCA tumour subtype cohort, as indicated. (C) Fraction of Genome Altered between iCCA and extrahepatic (including perihilar) CCA. (D) Log2 ratio plots of read count per bin, for three FFPE samples (top panels are tumour samples, lower panels are adjacent normal bile duct samples for each patient). (E) Images of the original slides samples, from which samples were excised.

To explore CNAs in CCA tumours, FFPE samples of tumour and adjacent normal bile duct tissue were obtained from 33 CCA patients from the liver fluke‐endemic region of northeastern Thailand (supplementary material, Table S1). DNA was extracted and shallow‐pass whole‐genome sequencing and DNA copy number calling were performed (see Materials and methods; supplementary material, Figures S1, S4, Table S2). We also subdivided the CNA analysis into extrahepatic, iCCA and perihilar samples to search for potential differences between subtypes (Figure 4A, supplementary material, Figure S5). Substantial variation was observed between CCA tumours in terms of CNA patterns (Figure 4A). As a cohort however, we noted a high degree of similarity in CNA landscapes between our tumour data and CNA data from iCCA from previous studies (e.g. [25, 26]) with common chromosomal losses in chromosomes 3p, 5q, 6q, 8p, 9p, 17p, 18q, and 21, and gains in chromosomes 1q, 3q, 5p, 7, 8q, 12p, 17q, and 19. We noted similar chromosomal alteration patterns between CCA tumour subtypes analysed in our study, although CNAs were often less obvious in our extrahepatic and perihilar tumour data due to lower sample numbers and a significantly lower CNA burden overall (Figure 4B,C).

Adjacent normal samples (from the nearby bile duct) were usually devoid of CNAs. However, we did note three normal samples with CNAs that also appeared robust upon manual review of the log2 ratios (Figure 4D). Two showed high level amplifications that were also present in the matched tumour sample, suggesting either metastatic spread of tumour cells to the nearby bile duct, or contamination during sample collection, although in these samples the tumour and sampled bile duct were distal to one another (Figure 4D,E), and both these patients were positive for metastasis (Figure 4A). One adjacent normal showed small amplifications in chromosome 10p that were not evident in the tumour (Figure 4D).

**Figure 5:**
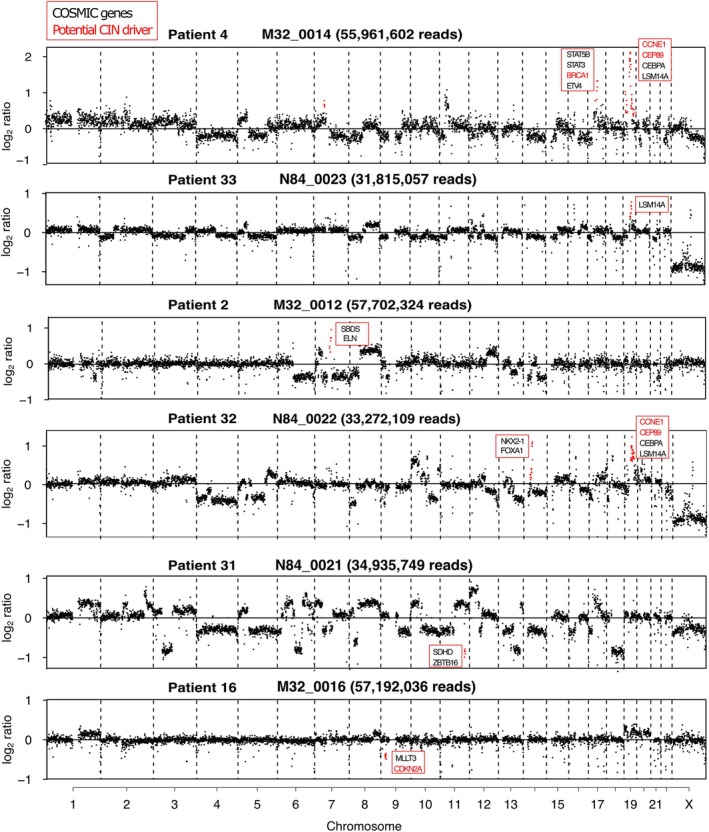
Log2 ratio plots from tumour FFPE samples indicating focal amplifications (amp) and deletions (del) (bins in amp or del marked in red). Genes within amp/dels were screened for the presence in the COSMIC cancer gene database [63], and genes present in COSMIC are indicated, with those putatively involved in chromosomal instability marked in red text.

We noticed that eight of the 33 tumour genomes exhibited focal amplifications (Figures 4D and 5). Focal amplifications have previously been explored in CCA, and may harbour functionally important cancer driver genes [27]. We identified the genes encompassed within these focal amplifications (Figure 5; supplementary material, Table S3). We noted that *MDM2* was one of 25 genes in the centromere‐proximal chromosome 12 amplification in Patient 25 (Figure 4D). *MDM2* amplifications have been observed in CCA patients [27, 60]. *KRAS* was also identified in the upstream focal amplification on chromosome 12. The focal amplifications on chromosome 7 in Patient 19 contained two cell cycle genes, *CDK6* and *CDK13*. *CDK6* is frequently amplified/overexpressed in multiple cancer types [61] and has been proposed as a targetable vulnerability in CCA [62]. In addition, we noted other focal amplifications or deletions that contained potentially relevant cancer driver genes, including two samples with a *CCNE1* amplification, and an unusual focal amplification of *BRCA1* in one of these patients (Figure 5; supplementary material, Table S3).

## Discussion

This is the first study to determine the presence of ongoing CIN in CCA, and to test for specific CIN driver mechanisms. We found that chromosome segregation errors were elevated in all three CCA cell lines, above what we observed in normal bile duct cells, or what has previously been reported in other normal, diploid cultured cell lines. We observed some potential differences between the cell lines. While all three CCA lines showed elevated levels of gH2Ax foci and ultrafine bridges, only KKU‐213A showed high levels of acentric fragments that we have previously associated with induction of DNA replication stress [18, 39]. By contrast, KKU‐M055 and KKU‐100 showed a higher incidence of chromatin bridges, potentially resulting from replication stress, faulty DNA repair or telomere deprotection. The overall rate of chromosome segregation errors was somewhat lower than that observed in other cancer types we have previously examined, such as colorectal and high‐grade serous ovarian carcinomas [18, 39] (Figure 1C).

We also performed single‐cell analyses to determine the extent of cell–cell heterogeneity caused by CIN using spectral karyotyping and single cell WGS. Both methods revealed a high level of cell–cell heterogeneity in CNAs, demonstrating that analysing bulk sequencing of tumours may obscure the extent of ongoing chromosomal instability. KKU‐M055 appeared more heterogeneous than KKU‐213A, despite both cell lines exhibiting similar rates of chromosome segregation errors. Since only very large (>~20 Mb) chromosomal alterations are likely to be visualised using microscopy, we conclude that KKU‐M055 may experience a higher rate of CIN, particularly involving smaller CNAs, or that other factors, such as differential tolerance to chromosome alterations, may explain the increased heterogeneity of KKU‐M055 cells.

We analysed the CNA incidence and patterns in 33 paired tumour and normal CCA samples from northeastern Thailand, a region endemic for CCA due to high levels of liver fluke contamination. Despite being from a liver fluke‐endemic area, we acknowledge that without ELISA testing, we cannot definitively verify liver fluke as the causative event of CCA in our patient samples. We observed a high variation in CNA burden between individual CCA tumours from different patients. However, as a cohort, the most frequent CNA patterns matched those previously reported (e.g. [26]). The highest copy number burden was in iCCA tumours compared to extrahepatic and perihilar CCA, although many of the specific alterations were similar between subtypes.

We noted that several CCA tumours harboured focal amplifications or deletions that contained relevant cancer driver genes, including *MDM2*, *KRAS*, and two samples with *CCNE1* amplification, and an unusual focal amplification of *BRCA1* in one of these patients (Figure 5, and supplementary material, Table S3). *CCNE1* amplification is common in high‐grade serous ovarian cancer and is proposed to drive elevated replication stress [64, 65]. *CCNE1* and *BRCA1* or *BRCA2* deletions or mutations are considered to be mutually exclusive in high‐grade serous ovarian carcinoma, due to an increased dependence on homologous recombination‐mediated DNA repair, in the presence of elevated replication stress [66]. Therefore, it is tempting to speculate that this *BRCA1* focal amplification represents a tolerance response to *CCNE1* amplification and high replication stress, although it remains unclear whether overexpression of *BRCA1* is sufficient to functionally rescue replication stress. It is also possible that the amplified *BRCA1* allele carries a mutation or indel as has been previously observed in a very‐low‐frequency of iCCAs [67]. A low proportion of CCAs (~3%) harbour mutations in *BRCA1*/*2* [68], and there is some evidence that Poly(ADP‐Ribose) polymerase (PARP) inhibition might be an effective therapeutic strategy for these patients (e.g. [69]). The finding that replication stress may be elevated in CCA, and perhaps be predicted by a genomic biomarker (i.e. *CCNE1* amplification), suggests that further research into replication stress as a targetable vulnerability in CCA could be warranted. For example, emerging replication stress response (RSR) inhibitors, such as ATR and Wee1 inhibitors currently undergoing clinical trials in other cancer types [53], may also be of therapeutic benefit in CCA from liver fluke‐endemic regions.

Lastly, we analysed adjacent normal tumour sections separately, in order to determine whether any CNAs were present. In two cases we could see evidence of high copy number focal amplifications (Figure 4D), suggesting that these samples could have contained migrating CCA tumour cells, although we acknowledge that contamination could also explain these results.

Overall, our study provides evidence that CIN is present in fluke‐associated CCA cell lines, and suggests a contribution of DNA replication stress. Although from a relatively small cohort, our study of CCA tumour samples also provides a deeper insight into focal amplifications in CCA from liver fluke‐endemic northeastern Thailand. Our analyses herein have stopped short of directly linking CIN mechanisms characterised in CCA cell lines with copy number alteration data from CCA tumours, due to a lack of knowledge of which copy number alteration features are caused by which CIN mechanism. Future work will focus on integrating information from *in vitro* and *in vivo* data to accurately predict CIN driver mechanisms and rates in order to derive novel therapeutic strategies.

## Author contributions statement

Cell biology, cytogenetics, and FFPE DNA extraction from tumours were carried out by RD, supervised by NS and SEM. Bioinformatics analysis was carried out by MAG, supervised by SEM. Single‐cell sequencing was performed by ILM, supervised by SEM. SW performed tissue sample identification. mFISH was performed by DM. The project was conceived by SEM, NS and RD. The article was written and edited by all authors.

## Supporting information


**Figure S1.** Cellularity analysis for FFPE whole genome sequencing samples
**Figure S2.** Multiplex FISH (mFISH) images from KKU‐M055 and KKU‐213A cell lines, used for analysis in Figure 2
**Figure S3.** Single cell genome sequencing profiles and pseudobulks
**Figure S4.** Images of the FFPE tumour slides used by pathologist to score and annotate tumour (T) and nearby normal bile duct (B) tissue
**Figure S5.** Heatmaps of CNAs derived from low pass whole genome sequencing from DNA extracted from FFPE tumour samples

**Table S1.** FFPE tumour information (provided as separate Excel file)

**Table S2.** CSV files (provided as separate Excel folder)

**Table S3.** Detailed list of focal amplifications and deletions identified in FFPE tumours (provided as separate Excel file)

## Data Availability

Whole‐genome sequencing and single‐cell genome sequencing data have been deposited at the EMBL‐EBI with the following accession number PRJEB87878 [71].
