# Supplementary material for: Chromosomal instability and genomic alterations in cholangiocarcinoma from Northeastern Thailand
Source: J Pathol. 2025 Sep 15;267(3):261–74. doi: 10.1002/path.6464 (PMC12531121; doi:10.1002/path.6464)
Supplement: Supplementary file 1 — Figure S1. Cellularity analysis for FFPE whole genome sequencing samples Figure S2. Multiplex FISH (mFISH) images from KKU‐M055 and KKU‐213A cell lines, used for analysis in Figure 2 Figure S3. Single cell genome sequencing profiles and pseudobulks Figure S4. Images of the FFPE tumour slides used by pathologist to score and annotate tumour (T) and nearby normal bile duct (B) tissue Figure S5. Heatmaps of CNAs derived from low pass whole genome sequencing from DNA extracted from FFPE tumour samples [file PATH-267-261-s001.docx]

**Chromosomal instability and genomic alterations in cholangiocarcinoma from Northeastern Thailand**

R Deenonpoe, MA Guscott *et al. J Pathol* <https://doi.org/10.1002/path.6464>

**Supplementary Figures S1–S5**

**Supplementary Tables S1–S4 (provided as separate Excel files)**


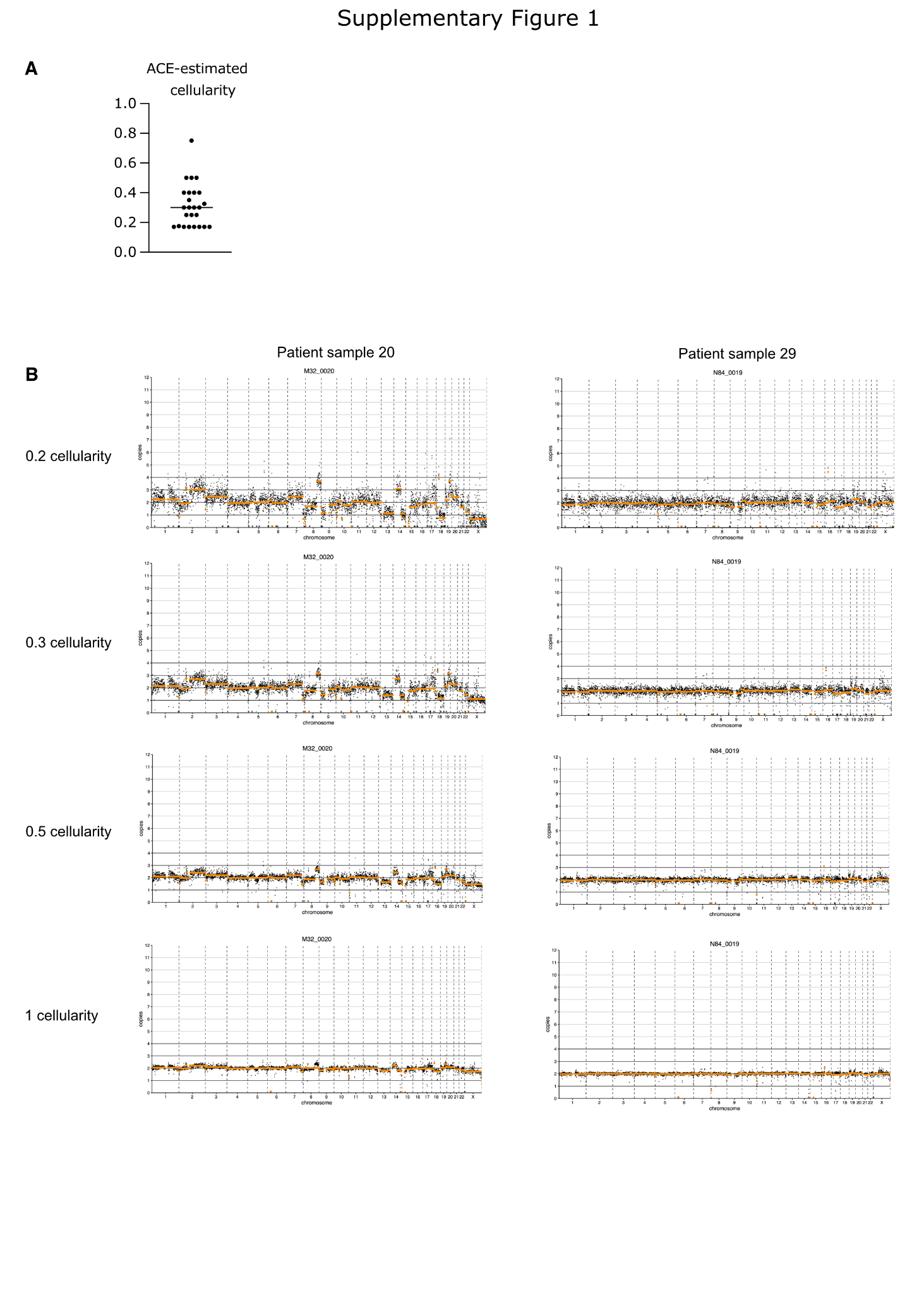


**Figure S1.** **Cellularity analysis for FFPE whole-genome sequencing samples.** (A) ACE-derived cellularity estimate for all FFPE tumour samples. (B) ACE-derived copy number plots derived from the cellularities shown.


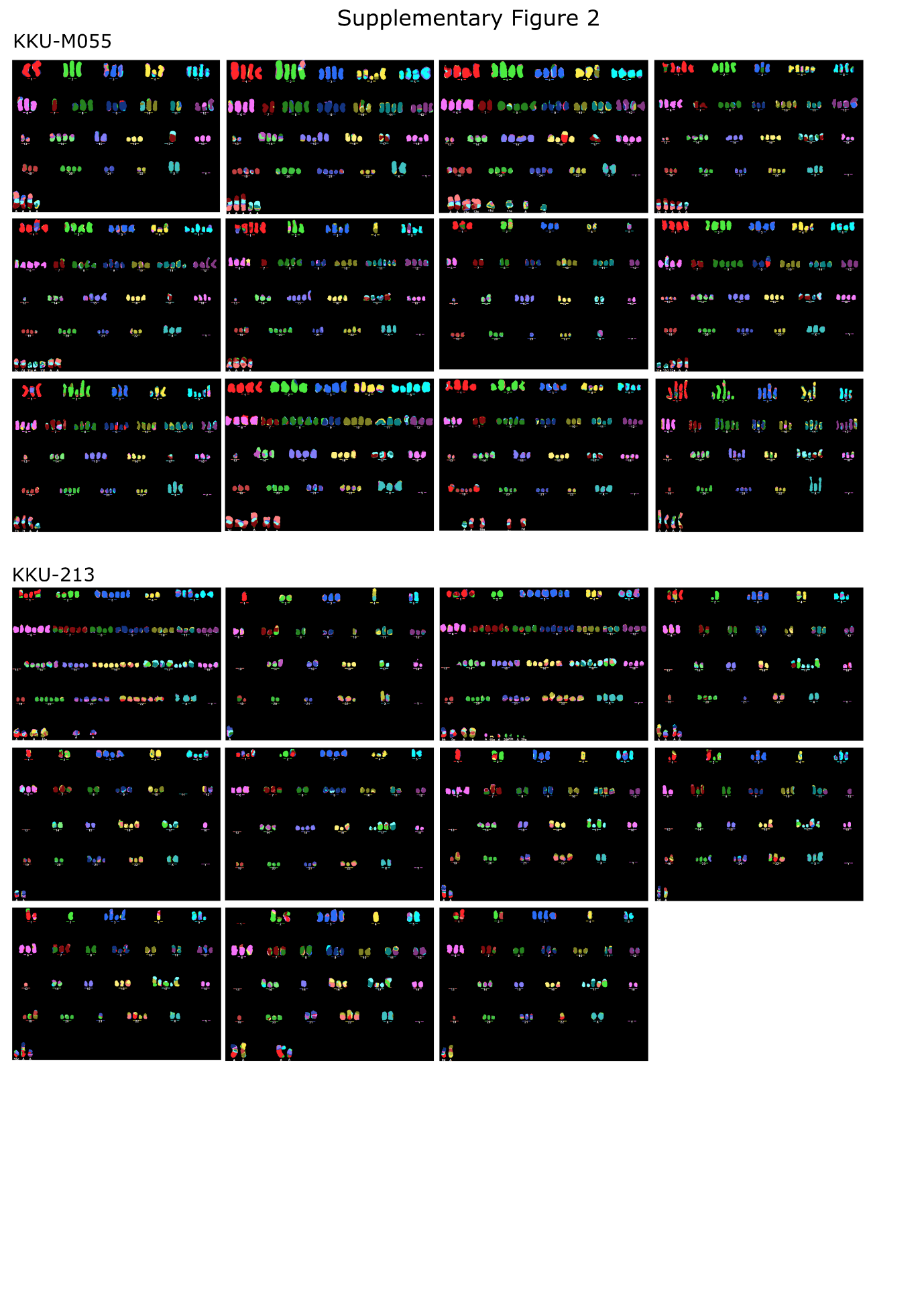


**Figure S2.** **Multiplex FISH (mFISH) images from KKU-055 and KKU-213A cell lines, used for analysis in Figure 2.**


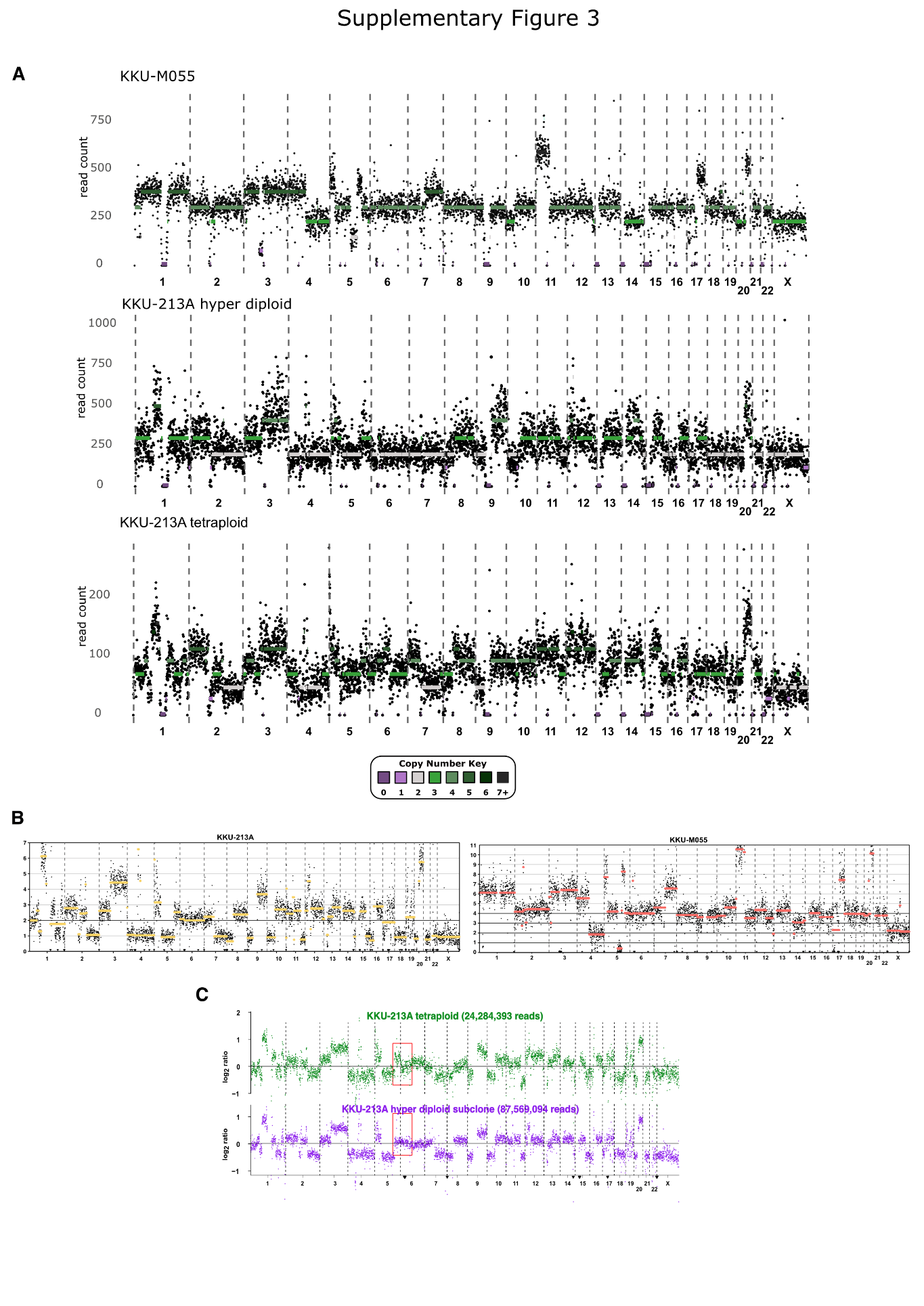


**Figure S3.** **Single-cell genome sequencing profiles and pseudobulks.** (A) Single-cell read count profile examples from KKU-M055 cells and representative tetraploid and hyperdiploid KKU-213A cells sequenced using DLP-plus. (B) Absolute copy number estimation of KKU-M055 and KKU-213A pseudobulks. (C) Psuedobulk log2 read count profiles of ploidy subclones identified in KKU-213A.


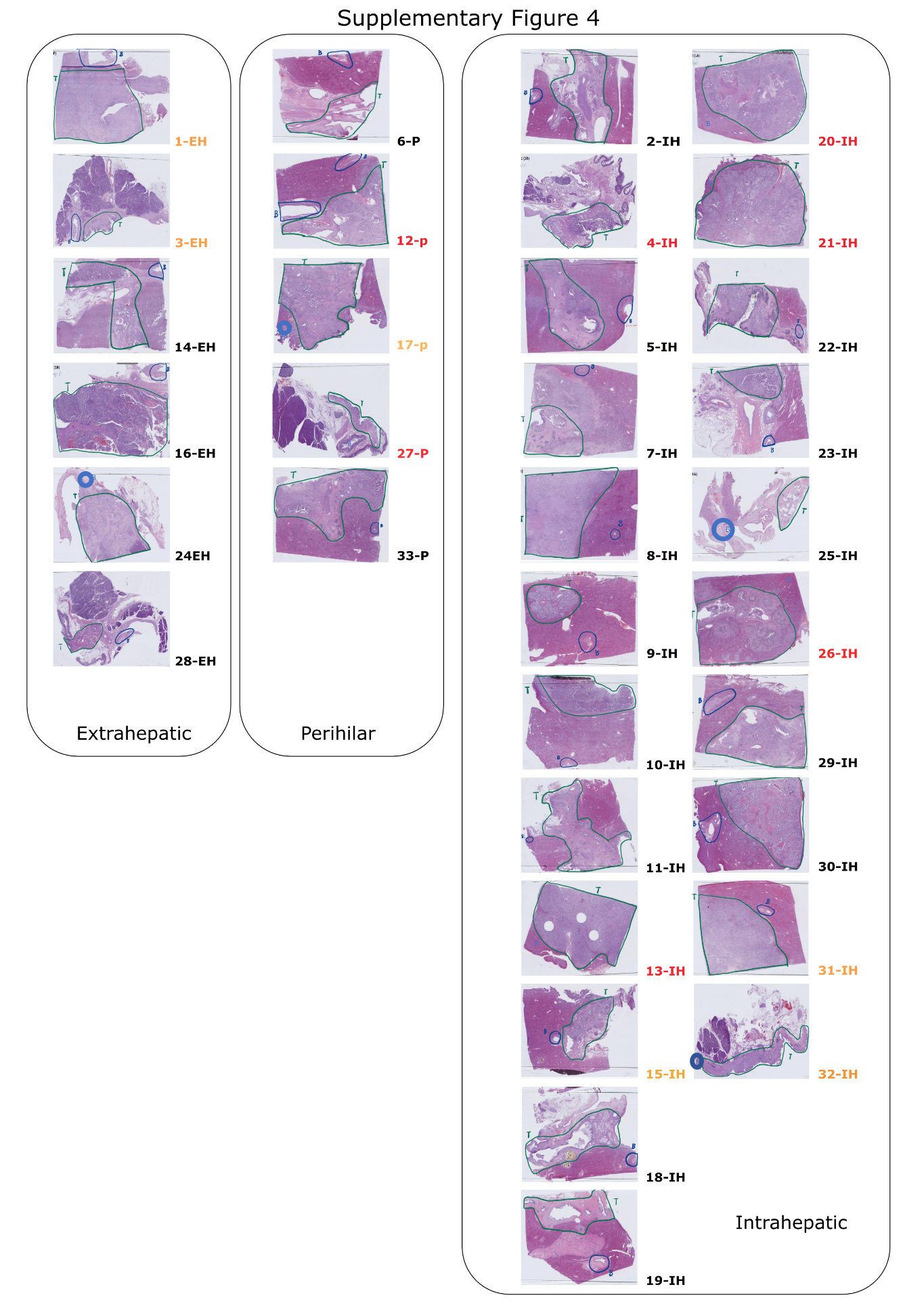


**Figure S4.** **Images of the FFPE tumour slides used by the pathologist to score and annotate tumour (T) and nearby normal bile duct (B) tissue.** Samples IDs are colour-coded for whether the normal bile duct was considered to be distant (black), moderately separated (yellow), or immediately adjacent (red) to account for the potential risk of contamination between tumour and normal tissue samples.


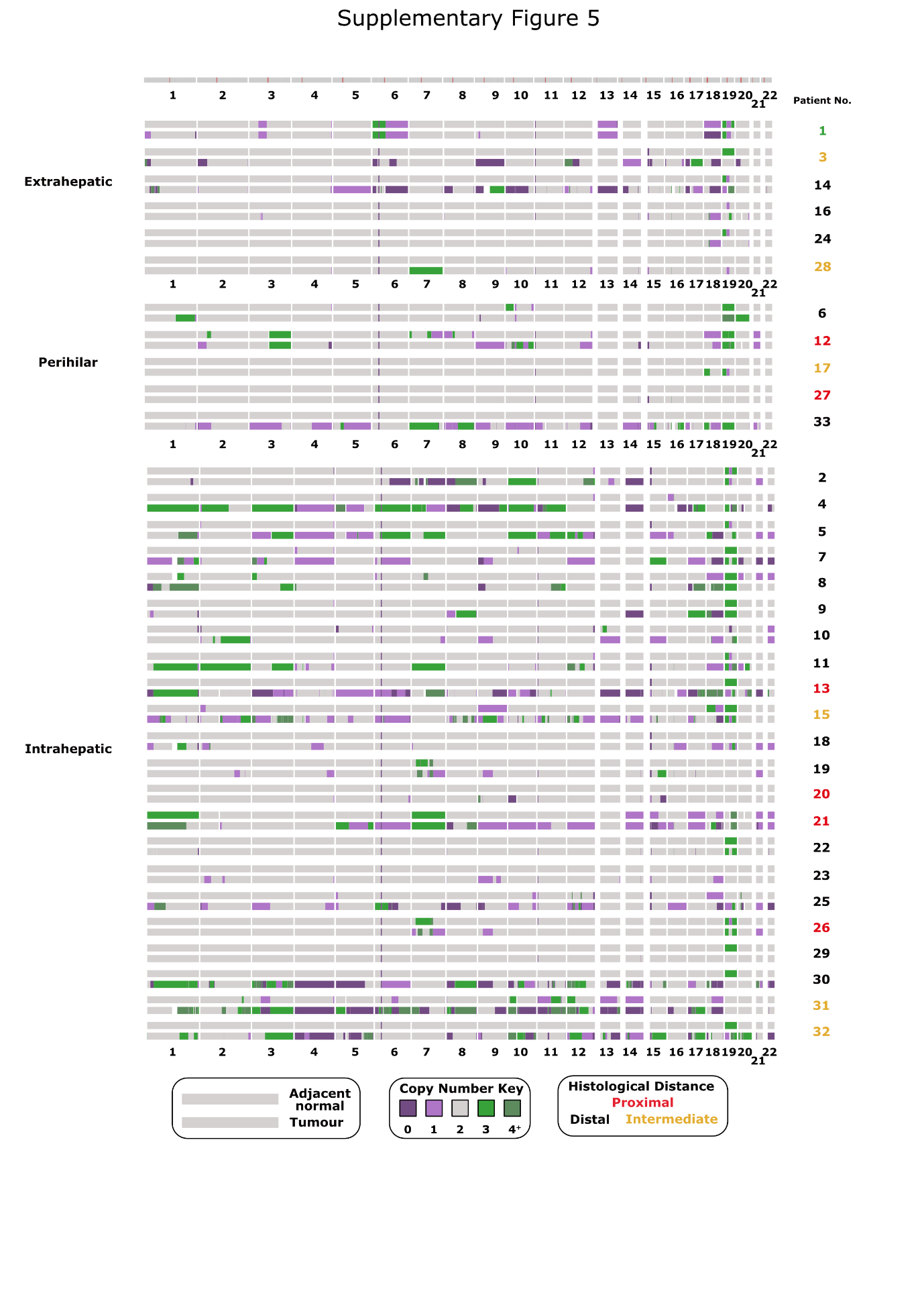


**Figure S5.** **Heatmaps of CNAs derived from low-pass whole-genome sequencing from DNA extracted from FFPE tumour samples.** For each sample, the top row represents normal bile duct tissue, the bottom row represents CCA tumour. Colour key indicates whether relative loss or gain of that part of the genome was detected. Samples are colour-coded for whether the normal bile duct was considered to be distant (black), moderately separated (yellow), or immediately adjacent (red) to account for the potential risk of contamination between tumour and normal tissue samples.
